# Application of Artificial Neural Networks (ANN) to Elucidate the Connections among Smell, Obesity with Related Metabolic Alterations, and Eating Habit in Patients with Weight Excess

**DOI:** 10.3390/metabo13020206

**Published:** 2023-01-30

**Authors:** Fernanda Velluzzi, Andrea Deledda, Mauro Lombardo, Michele Fosci, Roberto Crnjar, Enzo Grossi, Giorgia Sollai

**Affiliations:** 1 Obesity Unit, Department of Medical Sciences and Public Health, University Hospital of Cagliari, 09124 Cagliari, Italy; 2 Department of Human Sciences and Promotion of the Quality of Life, San Raffaele Roma Open University, 00166 Rome, Italy; 3 Department of Biomedical Sciences, University of Cagliari, 09042 Monserrato, Italy; 4 Autism Research Unit, Villa Santa Maria Foundation, 22038 Tavernerio, Italy

**Keywords:** metabolism, data mining, body weight, diet, Mediterranean, machine learning, feeding behaviour, olfaction, Sniffin’ Sticks, nutrition, overweight

## Abstract

Obesity is a severe health problem linked to an increased risk of comorbidity and mortality and its etiopathogenesis includes genetic, epigenetic, microbiota composition, and environmental factors, such as dietary habits. The olfactory system plays an important role in controlling food intake and meal size, influencing body weight and energy balance. This study aims to identify the connection between olfactory function and clinical and nutritional aspects related to weight excess in a group of 68 patients with overweight or obesity. All participants underwent the evaluation of olfactory function, anthropometric data (weight, height, BMI, waist circumference), clinical data (hypertension, disglycemia, dyslipidemia, metabolic syndrome), and adherence to the Mediterranean diet (Mediterranean Diet Score). A fourth-generation artificial neural network data mining approach was used to uncover trends and subtle associations between variables. Olfactory tests showed that 65% of patients presented hyposmia. A negative correlation was found between olfactory scores and systolic blood pressure, fasting plasma glucose, and triglycerides levels, but a positive correlation was found between olfactory scores and the Mediterranean diet score. The methodology of artificial neural networks and the semantic connectivity map “Auto-Contractive Map” highlighted the underlying scheme of the connections between the variables considered. In particular, hyposmia was linked to obesity and related metabolic alterations and the male sex. The female sex was connected with normosmia, higher adherence to the Mediterranean diet, and normal values of blood pressure, lipids, and glucose levels. These results highlight an inverse correlation between olfactory skills and BMI and show that a normosmic condition, probably because of greater adherence to the Mediterranean diet, seems to protect not only from an excessive increase in body weight but also from associated pathological conditions such as hypertension and metabolic syndrome.

## 1. Introduction

The sense of smell provides critical information on the environment and plays a relevant role in various aspects of the individual’s life: safety and survival, eating habits, social interaction, and quality of life [1,2,3,4]. Subjects with an impaired sense of smell report perceiving less palatable foods and experiencing less gratification in eating [5]. As a result, these people change their eating habits and try to compensate for reduced sensory gratification by adding spices and seasonings to foods and by preferring sweet and high-fat foods rather than healthier, but less palatable foods such as fruits and vegetables [5,6]. Among humans, there is a great inter-individual variability of olfactory function, ranging from normosmia (normal olfactory capacity) to anosmia (defined as total or specific olfactory blindness for some odors), passing through hyposmia (reduced olfactory ability) [1,7,8,9,10]. The causes of these alterations can be genetic [11,12,13,14,15], environmental [16,17,18,19], metabolic [20,21], and physiological [22,23,24,25]. As regards the physiological causes, an aspect of particular interest and still a matter of debate is represented by the differences in olfactory function between females and males. Although it is commonly accepted that females perform better than males, some studies do not report any sex-related differences [26,27]. Cognitive [24,28,29], social [24,29], and neuroendocrine factors have been suggested [30,31] as factors involved in supporting the better olfactory performance of females compared to males. Moreover, a recent study has highlighted the presence of possible genetic factors on the basis of the different olfactory performances related to sex [32].

Olfactory deficits are also associated with numerous chronic diseases, among which we can list hypertension, Parkinson’s and Alzheimer’s neurodegenerative diseases, depression, autoimmune/inflammatory diseases, diabetes, and obesity [33,34,35,36,37,38,39,40,41,42,43]. The metabolic changes associated with obesity, particularly circulating levels of hormones such as leptin, insulin, and ghrelin, seem to negatively affect the olfactory function of individuals [20,44,45,46]. On the other hand, by considering that the excessive consumption of foods rich in refined sugars and saturated fatty acids is one of the factors contributing to the development of obesity, at the expense of healthier foods rich in vitamins, minerals, and fibers, an alteration of olfactory function could favor the choice of the former type of food and can therefore be considered among the causes of obesity [47,48,49].

Overweight and obesity are chronic metabolic diseases affecting people of both sexes and all ages, with an increasing rate worldwide [50]. In the particular case of visceral adiposity, they are associated with an increased risk of cardiovascular and metabolic morbidity and mortality [51]. The biological mechanisms linked to the excess of visceral fat, among which low-grade chronic inflammation, oxidative stress, and altered adipokine pattern, also favor the development of other pathological conditions such as osteoarticular [52], respiratory [53], dermatological [54] diseases, or some types of cancer [55]. Furthermore, people with overweight or obesity frequently show sleep [56] and psychological disturbances [57] which in turn could worsen the outcome of obesity. The pathogenesis of obesity is complex, involving genetic, epigenetic, and environmental factors [58]. Among the latter, a hypercaloric, nutritionally poor diet, and sedentary behaviors play a key role in determining a persistent imbalance between energy intake and expenditure [59]. Consequently, lifestyle interventions characterized by changes in nutrition and increased physical activity represent a fundamental tool in the management of obesity [60]. As regards the dietary approach, the Mediterranean Diet (MD), consisting of a high consumption of food of vegetable origin and monounsaturated fats and a low consumption of red or processed meat and saturated fats, is known to have a protective effect against mortality and several chronic non-communicable diseases such as cardiovascular or neurodegenerative diseases, cancer, type 2 diabetes mellitus and obesity [61,62,63]. MD has also been shown to positively modulate the gut microbiota composition in patients with obesity [64], and an association between high adherence to MD and a beneficial microbiota pattern has been reported in extremely long-lived individuals [65]. However, despite the numerous reported health benefits, adherence to MD is progressively lowering, especially among young people in favor of a globalized diet based on packaged processed food rich in simple sugar and saturated fats [66].

Based on these considerations, the purpose of this work is divided into three objectives: (1) to assess the incidence of olfactory dysfunction in individuals with excess body weight; (2) to evaluate the presence of correlations between the olfactory function and the metabolic alterations associated with obesity; (3) to identify the connections between excess body weight and olfactory function, using an innovative data-mining approach with a fourth-generation Artificial Neural Network (ANN) analysis, a computational adaptive system capable of highlighting and identifying subtle trends and associations between variables. By means of this approach, the goal was to provide more information on the complex relationship between olfactory dysfunction and body weight and metabolic alterations associated with obesity, especially regarding sex-related differences. Therefore, this study will aim to highlight the positive and/or negative correlations between olfactory function and metabolic alterations associated with obesity; subsequently, an artificial neural networks (ANN) analysis and an innovative data mining analysis will allow us to understand natural processes, recreating these processes using automated models, and to discover hidden trends and associations among variables.

## 2. Materials and Methods

### 2.1. Participants

Sixty-eight outpatients (51 women, 17 men; age 54.87 ± 1.76 yrs) with overweight or obesity registered at the Obesity Unit of the University Hospital of Cagliari (Sardinia, Italy), hereafter referred to as OC patients, agreed to take part in this study. Exclusion criteria were pregnancy or lactation, the presence of head trauma, history of cancer, sinusitis or nasal sept disorders, and neurological or psychiatric diseases. Patients who asserted to have had nasal congestion or allergic reactions before undergoing the smell tests were excluded. All subjects were required to fast for at least 2 h prior to testing and to be fragrance-free.

All participants underwent the evaluation of anthropometric data (height, body weight, BMI, waist circumference), clinical data (hypertension, diabetes, dyslipidemia, metabolic syndrome), adherence to the Mediterranean diet (Mediterranean Diet Score, MDS), and olfactory function by means of the “Sniffin’ Sticks” test (as described in the next paragraph). As for the MDS, we also reported the mean score obtained in a control group of adult people (50 M, 50 F, age range 18–89 yrs) from the general population of the same geographical area (Sardinia, Italy).

The anthropometric evaluation was performed by the same expert nutritionist according to the current methodology [67]. Height, expressed in centimeters (cm), was measured using a wall-mounted stadiometer (SECA, Hamburg, Germany) on barefoot patients with the head being in the “Frankfort plane”. Body weight expressed in kilograms (kg) was measured by means of an impedance scale (TANITA BC420MA, Amsterdam, The Netherlands) while patients were in a fasting state and wearing only light clothes. The BMI was calculated through the ratio between the weight and the square of height (kg/m^2^) and was used to classify the weight status of the subjects: a BMI value ≥ 25 kg/m^2^ indicates overweight, while a BMI value ≥ 30 kg/m^2^ defines obesity. The waist circumference (WC), expressed in cm, was measured according to the NIH protocol (NIH, 2008), considering the International Diabetes Federation (IDF) cut-off values for the European population (94 cm for men and 80 cm for women) [68]. 

The clinical evaluation includincludeded the measurement of Sistolic and Diastolic Blood Pressure (S-BP and D-BP), Fasting Plasma Glucose (FPG), HDL cholesterol (HDL-C), and triglycerides (TG) values. The BP measurement was performed by the endocrinologist during the physical examination according to a standardized procedure [69]. The assessment of the metabolic variables consisted of a 12 h fasting blood sample for determination with standard methods of fasting plasma glucose (FPG), HDL cholesterol (HDL-C), and total triglycerides (TG) values. As for WC, also for all the aforementioned clinical variables we considered the IDF cutoff values, and Metabolic Syndrome (MS) was defined by the presence of a WC value higher than the sex-specific cut-off plus at least 2 of FPG ≥ 100 mg/dl or specific treatment for diabetes mellitus; TG ≥ 150 mg/dl; HDL-C < 40 mg/dl males, <50 mg/dl females or specific treatment for dyslipidemia; SBP and DBP ≥ 130/85 mmHg or specific treatment for hypertension [68].

The adherence to the Mediterranean Diet was evaluated by means of a validated and standardized questionnaire which allows the establishment of the degree of adherence through a score (Mediterranean Diet Score, MDS) in the range of 0–55. More specifically, the questionnaire considers the monthly or weekly consumption of 11 food groups, and assigns an increasing score from 0 to 5 for the consumption of foods typical of the Mediterranean model (unrefined cereals, potatoes, vegetables, fruit, legumes, fish, and olive oil), and a decreasing score from 5 to 0 for the consumption of foods considered far from the same model (meat or derivatives, poultry, whole dairy products); as for alcohol consumption, decreasing scores from 5 to 1 indicate a consumption <300 mL/day, <400 mL/day, <500 mL/day, <600 mL/day, respectively, while a score of 0 corresponds both to a consumption >700 mL/d or no consumption. The total score, obtained from the sum of the 11 individual scores, ranges from 0 to 55, with higher values indicating a higher adherence to MD [70]. Due to the lack of a specific cutoff, we considered the MDS of 30, corresponding to the mean value obtained in the control group from the general population, as a reference value of adherence to the MD. Table 1 shows the mean values ± ES of the anthropometric and clinical data, Mediterranean Diet Score and olfactory scores of the patients enrolled in the study considered separately by sex.

### 2.2. Olfactory Sensitivity Screening

The “Sniffin’ Sticks” test battery (Burghart Instruments, Wedel, Germany) was used to assess the individual orthonasal olfactory function. This method is validated in the health field and internationally credited. In fact, evaluation of the olfactory threshold (*t*-test), discrimination (D-test), and identification (I-test) are generally applied for olfactory screening [71]. Felt-tip pens were used to present odors: after removing the cap, the pen tip is placed for 3 s about 2 cm away from the nostrils. 

The olfactory threshold was established by means of 16 triplets. Each triplet consists of a pen containing an increasing concentration of n-butanol and two pens containing a solvent. The experimenter presents the triplets in increasing order till the test subject recognizes the pen with n-butanol in the same triplet twice in a row. This represents the starting position and the first reversal, i.e., the point at which the triplets are presented in descending order of n-butanol dilution. Each time the subject fails to recognize the target pen twice in the same triplet, the dilution order in which the triplets are displayed is reversed. The threshold score is the average of the last four of the seven reversals. To evaluate the odor discrimination, each triplet of the 16 used by the experimenter, is composed of two pens filled with the same odor and one soaked with a different one (target pen). The objective is to recognize the target pen and the number of correct answers represents the score obtained by the individual. Finally, 16 pens containing odors familiar to the subjects were used to ascertain the ability to identify them. For each pen, the participant has to choose among four possibilities and the number of correct identifications represents the score obtained.

Information about the participants’ olfactory scores, age, gender, height, weight, and BMI of the participants are recorded by the experimenter on a protocol during the test. The sum of the scores obtained with the *t*-test, D-test, and I-test gives the total TDI. This TDI score, as well as the scores obtained with *t*-test, D-test, and I-test, help classify subjects by their olfactory performance as normosmic or hyposmic [72].

### 2.3. Data Analysis

The results for continuous variables were expressed as mean value and standard error (M ± ES), while the results for categorical variables were expressed as absolute numbers and percentages. The comparison of the olfactory status between males and females was performed by means of the Fisher exact test, while the relationship between the continuous variables was evaluated using Pearson’s correlation test. For all the tests a *p*-value *p* < 0.05 was considered as the significance limit. The analyses were performed with GraphPad Prism 6 (GraphPad Software, San Diego, CA, USA).

**Figure 1:**
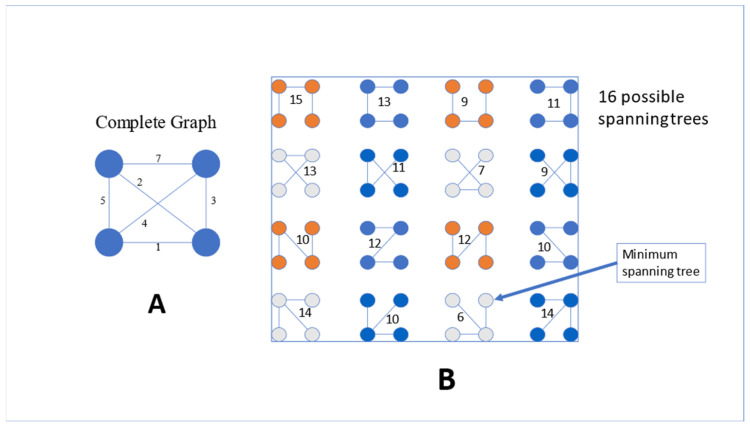
Minimum spanning tree: (**A**) a complete graph in which all points are connected and (**B**) 16 possible spanning trees.

The analyses were carried out using a fourth-generation artificial neural network (ANN) data mining approach. Using a mathematical strategy based on ANNs, Auto Contractive Map (Auto-CM) is a mapping method that can calculate the multidimensional association strength of each variable with all other variables in a dataset. Auto cm is very efficient at pointing out any form of recurring patterns, regular correlations, hidden trends, and associations between variables. In fact, this approach may create and construct a semantic connectivity map that preserves nonlinear relationships between variables, captures elusive connection schemes between clusters, and emphasizes intricate similarities between variables. ANNs aim to understand natural processes and recreate them using automated models, and have been used successfully in many fields of medicine to understand non-linear relationships among variables. This is useful when conclusive evidence is lacking on these associations due to intrinsic methodological limitations of standard statistical techniques in describing nonlinear and complex associations typically observed in biological systems. In recent years, these limits have been overcome by the introduction of artificial neural networks (ANN) analysis and an innovative data mining analysis known as auto-contractive map (AutoCM) based on ANN architecture: AutoCM allows to uncover hidden trends and associations among variables, using a fuzzy clustering approach. The added value of this approach in the study of biological systems is represented by its ability to highlight the organizational principles of a network of variables and therefore to map biological processes using automatic and analytical models to reconstruct the imprecise, nonlinear, and simultaneous pathways underlying a complex set of data. In the last decade, AutoCM has been successfully tested across the medical field. Recently, a machine learning model has been created and used to predict with high precision the taste function of subjects [73]. The description of the 3-layer architecture and the mathematical models of Auto-CM is reported in Buscema and Grossi [74]. This model has a training phase and a learning phase. To put it in non-technical terms, Auto-CM chooses the “weights” of the vector matrix, which describe the dataset warped landscape and allows for a direct interpretation. In fact, these weights may be easily visualized by converting them into physical distances: variables with greater connection weights move relatively closer to one another, and vice versa. The distances are proportional to the importance of many-to-many relationships across all variables. A graph known as a “semantic connectivity map” is produced by applying a mathematical filter, the minimum spanning tree, to the matrix of distances [75,76]. This approach enables a visual mapping of the intricate network of connection patterns between variables, making it easier to identify the variables that are crucial for understanding the graph. The dimensionality issue is solved by the adaptive learning inference algorithms, which are based on the idea of a functional estimation similar to ANNs. Due to this, we opted against using the Bonferroni adjustment (which is used when significance tests are conducted with dependent variables) and instead used an exploratory approach to investigate significant connections among numerous independent factors. The Minimum Spanning Tree (MST) displays the shortest combination out of all potential methods to connect the variables in a tree, as shown by the example in Figure 1.

Figure 1 shows the graph theory applied to four points (variables) having in multidimensional space the distances visible on the arches. Figure 1A depicts a complete graph in which all points are connected. Figure 1B describes 16 possible spanning trees, i.e., the possibilities to connect the four points avoiding loops. By considering the distances there is one spanning tree in which the sum of distances produces the shortest path (sum = 6). This is the minimum spanning tree of this set of points.

The Auto-CM, which is based on the MST theory, provides a graph in which the distances between variables indicate their bonding strength (weights), revealing links between variables [74,77,78]. Practically speaking, MST demonstrates the shortest possible combination that displays the data in a condensed graph as well as the optimum approach to connect the variables in a tree. This method offers a diagram showing the relationships between the main hubs of the system and the variables. Hubs are variables in the map that have the greatest number of connections. The initial weights are not posed at random by the Auto-CM. The Auto-CM, on the other hand, begins with the same value. As a result, the generated graph can be replicated throughout numerous runs. In other words, the graph simply recognizes the pertinent associations, arranging them into a logical whole, while the Auto-CM visualizes in space the connections among variables (“closeness”). The inner node that is left after the “leaves” nodes are removed by bottom-up recursively pruning is known as the “central node.” The “nervous system” of any data set can be represented by the MST. In fact, the total energy of the system is obtained by adding the connection strengths of all variables. The MST selects just the connections that minimize this energy, that is, only those really necessary to make the system coherent.

## 3. Results

### 3.1. Anthropometric and Metabolic Variables

The anthropometric evaluation allowed us to classify the patients who took part in the study as overweight or obese subjects on the basis of their BMI value. In detail, 22/68 patients (16 F, 6 M) were overweight, while 46/68 patients (35 F, 11 M) presented obesity (Table 2).

The clinical evaluation made it possible to assess the presence of metabolic alterations obesity-related such as hypertension, hyperglycemia, and abnormal HDL and TG levels. In particular, as shown in Table 3, we found that 41/68 patients (29 F, 12 M) had hypertension, 22/68 patients (15 F, 7 M) had hyperglycemia, 17/68 patients (15 F, 2 M), and 16/68 patients (11 F, 5 M) showed low HDL and high TG levels, respectively. In addition, according to the above-mentioned IDF criteria (Alberti, 2005), 27/68 patients (20 F, 7 M) showed metabolic syndrome, while 41/68 patients (31 F, 10 M) did not meet the diagnostic criteria for metabolic syndrome, as shown in Table 3. Finally, considering the score obtained with the Mediterranean Diet Questionnaire, we assessed the patients’ adherence to the Mediterranean Diet. The results shown in Table 3 highlight that 38/68 patients (30 F, 8 M) scored ≥ 30, while 30/68 patients (21 F, 9 M) scored less than 30, indicating a higher or lower adherence to MD, respectively.

### 3.2. Olfactory Function and Correlation Analysis

The distribution of subjects classified as normosmic and hyposmic based on their overall TDI olfactory status and individually for their T, D, or I olfactory status is shown in Table 4. In detail, based on TDI scores, 44/68 patients were classified as hyposmic and 24/68 as normosmic. Instead, based on the score obtained from each subtest, 20/68 patients were classified as hyposmic and 48/68 as normosmic by means of T olfactory score, 28/68 patients were classified as hyposmic and 40/68 as normosmic by means of D olfactory score. Finally, 34/68 patients were classified as hyposmic and 34/68 as normosmic by means of I olfactory score. None were classified as anosmic.

Table 5 shows the distribution of female and male patients classified by their olfactory status. The percentage of female patients classified as normosmic or hyposmic by their TDI olfactory status differed from that determined for male patients (χ2 = 8.5859, *p* = 0.0034) (Table 5). In detail, 45.10% (n = 23/51) and 54.90% (n = 28/51) of females were, respectively normosmic or hyposmic, while 5.88% and 94.12% of males were normosmic or hyposmic, respectively. Table 5 also shows the distribution of female and male patients classified as normosmic or hyposmic based on their Threshold (T), Discrimination (D), and Identification (I) olfactory status. Fisher’s method showed that the percentage of females classified as normosmic or hyposmic for their I olfactory status differed from that of male patients (χ2 = 5.6717, *p* = 0.0172). Specifically, 56.86% (n = 29/51) and 43.14% (n = 22/51) of females were, respectively normosmic or hyposmic, while in the case of male patients, 29.41% were classified as normosmic and 70.59% as hyposmic. No differences were found in distribution between subjects classified as normosmic or hyposmic on the basis of T and D olfactory status.

**Figure 2:**
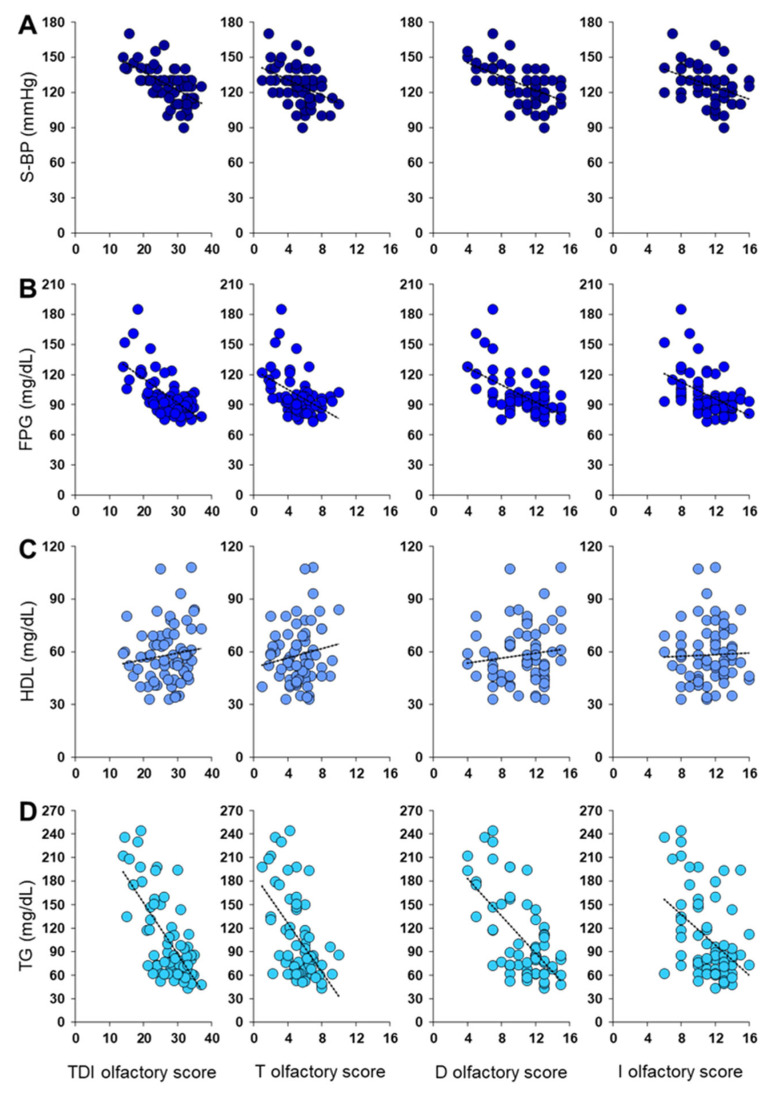
Correlation analysis between olfactory scores obtained by each patient and her/his (**A**) Systolic Blood Pressure (S-BP), (**B**) Fasting Plasma Glucose (FPG), (**C**) HDL levels, and (**D**) triglycerides levels (TG).

Figure 2 shows the correlation results between olfactory scores and S-BP, FPG, HDL, and TG plasma levels. Pearson’s correlation test highlighted a negative correlation between the TDI olfactory score obtained by each patient and her/his S-BP, FPG, and TG levels (Pearson’s r > −0.59, *p* < 0.0001). The same negative correlations were also found between S-BP, FPG, and TG levels of each patient and her/his T (Pearson’s r > −0.42, *p* < 0.0005), D (Pearson’s r > −0.55, *p* < 0.0001) and I (Pearson’s r > −0.40, *p* < 0.001) olfactory scores. Instead, no correlation was found between each patient’s olfactory score and her/his HDL levels (Pearson’s r < 0.15, *p* > 0.21).

**Figure 3:**
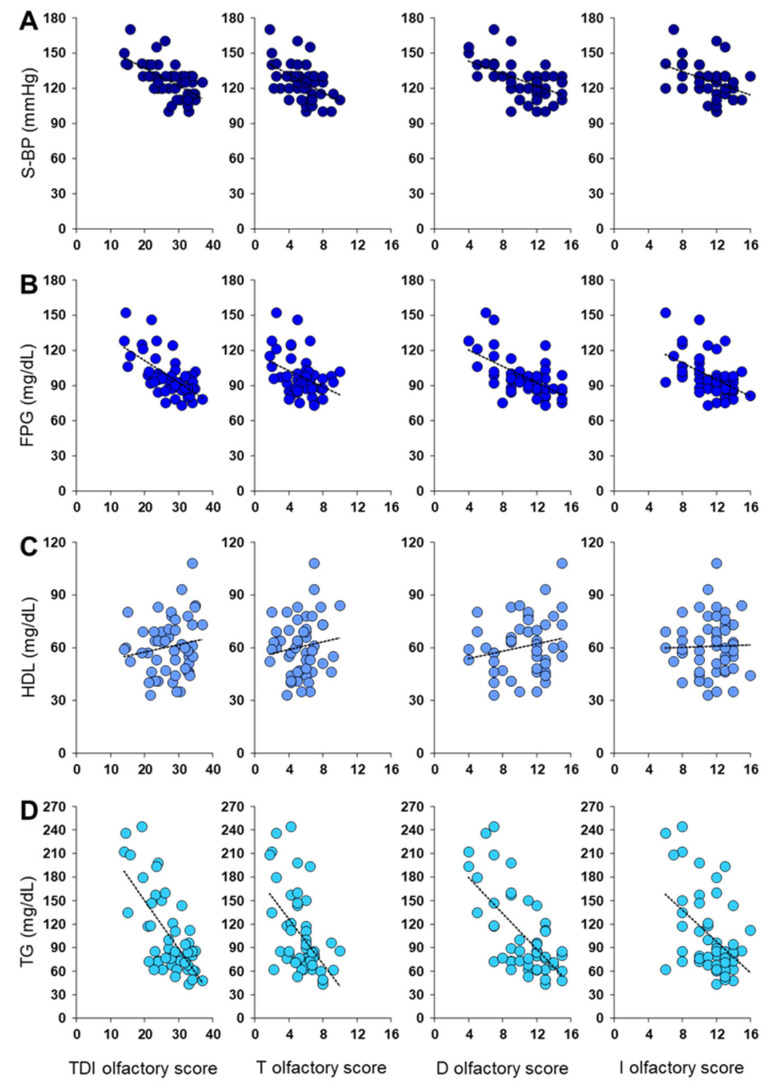
Correlation analysis between olfactory scores obtained by each female patient and her (**A**) Systolic Blood Pressure (S-BP), (**B**) Fasting Plasma Glucose (FPG), (**C**) HDL levels, and (**D**) triglycerides levels (TG).

Figure 3 shows a negative correlation between olfactory scores obtained by each female patient and her S-BP (Pearson’s r > −0.39, *p* < 0.005), FPG (Pearson’s r > −0.39, *p* ≤ 0.005), and TG levels (Pearson’s r > −0.46, *p* < 0.001). No correlation was found between each female’s olfactory score and her HDL levels (Pearson’s r < 0.20, *p* > 0.16).

**Figure 4:**
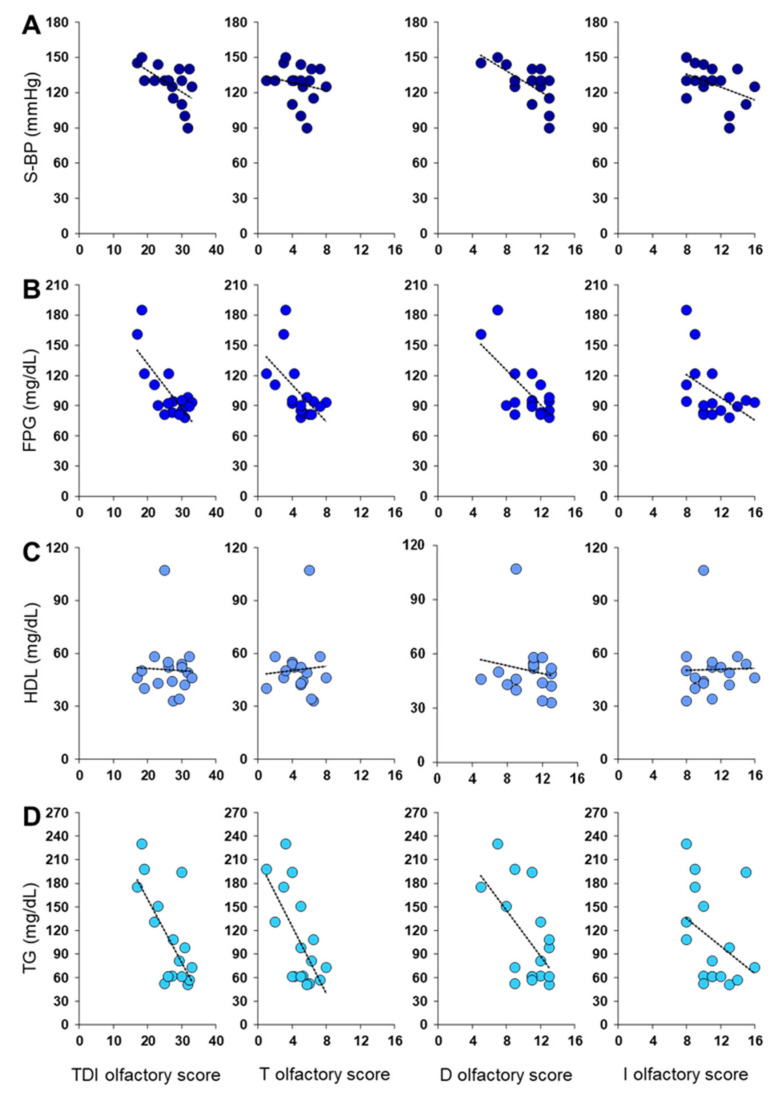
Correlation analysis between olfactory scores obtained by each male patient and his (**A**) Systolic Blood Pressure (S-BP), (**B**) Fasting Plasma Glucose (FPG), (**C**) HDL levels, and (**D**) triglycerides levels (TG).

Figure 4 shows a negative correlation between TDI and D olfactory scores obtained by each male patient and his S-BP (Pearson’s r > −0.58, *p* ≤ 0.015), FPG (Pearson’s r > −0.68, *p* < 0.005), and TG levels (Pearson’s r > −0.58, *p* ≤ 0.016). Instead, in the case of the T olfactory score, the results showed a negative relationship only with FPG (Pearson’s r = −0.56, *p* = 0.019) and TG levels (Pearson’s r = −0.66, *p* = 0.004). Finally, no correlation was found between each male’s olfactory score and his HDL levels (Pearson’s r < 0.16, *p* > 0.55).

**Figure 5:**
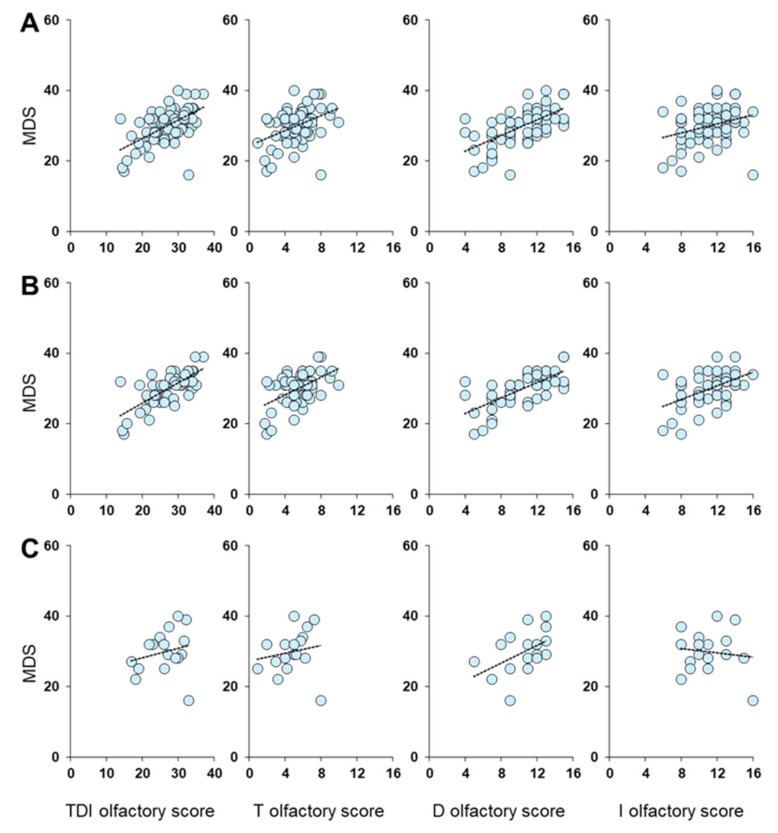
Correlation analysis between olfactory scores obtained by each patient and his/her Mediterranean Diet Score (MDS) in the whole sample (**A**), and in females (**B**) and males (**C**), separately.

A positive correlation was found between the Mediterranean Diet Score (MDS) obtained by each patient and her/his TDI (Pearson’s r = 0.58, *p* < 0.0001), T (Pearson’s r = 0.40, *p* < 0.001), D (Pearson’s r = 0.62, *p* < 0.0001), and I (Pearson’s r = 0.30, *p* = 0.012) olfactory score (Figure 5). When patients were divided according to sex, Pearson’s correlation test showed a positive correlation in the case of females, for all olfactory scores considered (Pearson’s r > 0.48, *p* < 0.001). Conversely, no correlation was found in the case of males (Pearson’s r < 0.48, *p* < 0.051).

### 3.3. Semantic Connectivity Map

**Figure 6:**
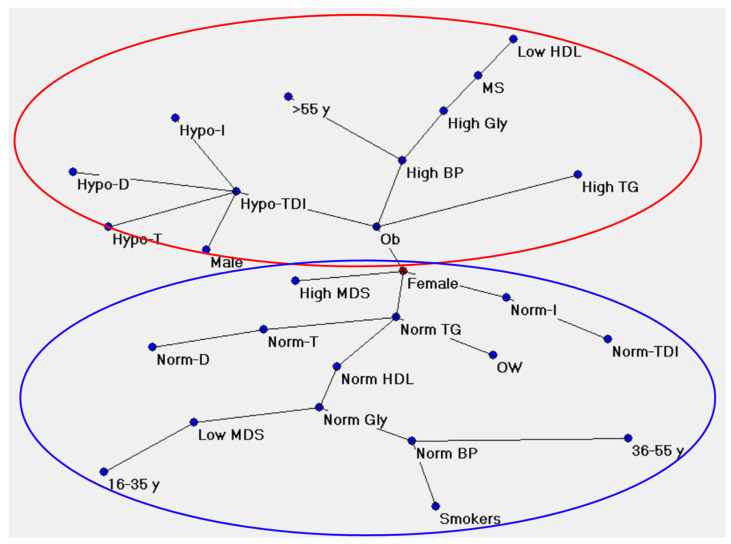
Map I shows the connections among variables in the unhealthy (red circle) or healthy (blue circle) form.

The semantic connectivity map (Auto-CM method)-MST graph (Map I in Figure 6) shows the connections between the variables considered and highlights that these are arranged to form a tree that can be divided into two parts: the unhealthy pattern of the variables appears in the upper part, while the healthy one appears in the lower one. In particular, it is noted that in the upper part of the graph, the variable “obesity” acts as a hub, a clinical condition having a considerable number of connections with the clinical, olfactory, anthropometric, and nutritional variables in the unhealthy area, while in the lower part of the tree the variable “norm TG” acts as a hub, which has a considerable number of connections with the same variables, but in the healthy and/or non-compromised area. In detail, map 1 (Figure 6) shows that obesity condition is strongly connected with olfactory dysfunction and male sex, hypertension, disglycemia, dislipidemia, metabolic syndrome, and older age. Instead, “norm TG” is connected with a normal olfactory function, healthy clinical parameters (blood pression, glycemia, HDL, and TG), and with adherence to the Mediterranean diet through the female node, marked in red since it is the central node of the graph.

Hypo-T, Hypo-D, Hypo-I, and Hypo-TDI: subjects classified as hyposmic based on their scores obtained with the Threshold (T), Discrimination (D), Identification (I) test, and their sum (TDI); Norm-T, Norm-D, Norm-I, Norm-TDI: subjects classified as normosmic based on their scores obtained with the Threshold (T), Discrimination (D), Identification (I) test and their sum (TDI); OW: subjects with overweight; Ob: subjects with obesity; High BP: high blood pressure; Norm BP: normal blood pressure; High Gly: high glycemia; Norm Gly: normal glycemia; High TG: high triglycerides; Norm TG: normal triglycerides; Low HDL: High-Density Lipoprotein; Norm HDL: High-Density Lipoprotein; MS: subjects with metabolic syndrome; High MDS: high adherence to the Mediterranean diet (score ≥ 30); Low MDS: poor adherence to the Mediterranean diet (score < 30).

**Figure 7:**
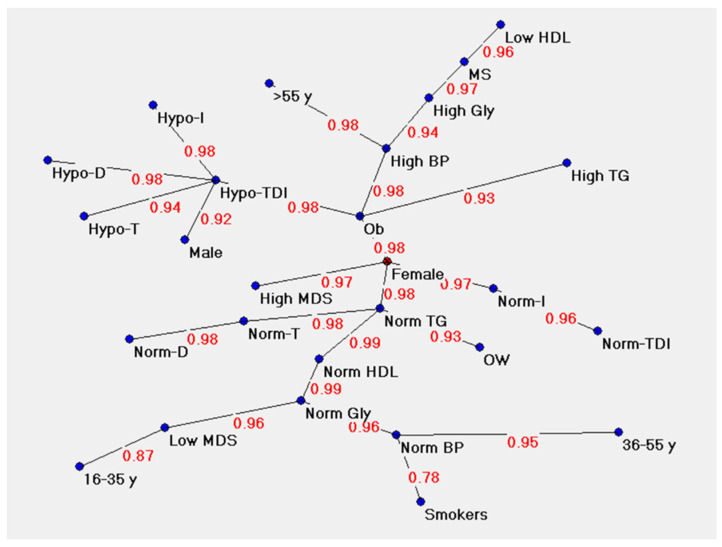
Map II shows the strength of the association among the variables considered.

Map II in Figure 7 shows the strength of the association between the variables considered: olfactory function, clinical parameters, anthropometric data, and eating behavior. The associations are visualized by the concept of “proximity”: the variables whose connection weights are greater are relatively close and vice versa. The strength of the relationship between variables progressively increases up to the value of 1 (the strongest connection level). In our maps, most of the link strength values were above 0.92, indicating a strong connection, and most of them were very close to 1. Only the connection between poor adherence to the Mediterranean diet and young age and that between normal blood pression and a cigarette smoking habit showed connection strength values of 0.87 and 0.78, respectively.

Hypo-T, Hypo-D, Hypo-I, and Hypo-TDI: subjects classified as hyposmic based on their score obtained with the Threshold (T), Discrimination (D), Identification (I) test, and their sum (TDI); Norm-T, Norm-D, Norm-I, Norm-TDI: subjects classified as normosmic based on their score obtained with the Threshold (T), Discrimination (D), Identification (I) test and their sum (TDI); OW: subjects with overweight; Ob: subjects with obesity; High BP: high blood pressure; Norm BP: normal blood pressure; High Gly: high glycemia; Norm Gly: normal glycemia; High TG: high triglycerides; Norm TG: normal triglycerides; Low HDL: High-Density Lipoprotein; Norm HDL: High-Density Lipoprotein; MS: subjects with metabolic syndrome; High MDS: high adherence to the Mediterranean diet (score ≥ 30); Low MDS: poor adherence to the Mediterranean diet (score < 30).

## 4. Discussion

One of the main functions of the olfactory system is to play a relevant role in the choice of food and in the eating behavior of individuals [4,5,79]. Changes in dietary habits and/or the desire for certain foods that characterize subjects who have problems with their sense of smell could affect body weight and metabolic control, even with long-term consequences [20,80].

Based on these considerations, the first goal of this work was to assess the incidence of olfactory dysfunction in a group of patients with overweight or obesity. The results we found show that approximately 65% of patients presented a reduced general olfactory function and that 30%, 40%, and 50% of them were classified as hyposmic for odor perception, discrimination, and identification skills, respectively. These data are in accordance with previous studies which showed that patients with excess body weight have a reduced olfactory function and that hyposmia increases with increasing BMI [41,49,81,82]. While obesity leads to imbalances in the circulating levels of peptides such as leptin, insulin, and ghrelin which can decrease or increase olfactory sensitivity [20,44,45,83], a compromised sense of smell can instead affect body weight by acting on the control mechanisms of eating behavior. In fact, the olfactory information can reach the lateral and ventromedial hypothalamic regions where hunger and satiety centers are located, respectively; in this way the olfactory system appears to control the activity of those neurons that regulate food intake and body weight [84]. Furthermore, some evidence suggests that higher order neurons in the orbitofrontal cortex are involved in the process of food-related gratification, thus contributing to satiety and consequently to meal termination [85,86]. Finally, the sense of smell participates in the responses of the cephalic phase of digestion, which are not only useful for preparing the body to digest, absorb and metabolize food, but also for the mechanisms that lead to starting and ending a meal [87]. In this regard, we looked for a correlation between olfactory function and eating habits, and the finding of a positive correlation between the olfactory scores obtained by the patients and their adherence to the Mediterranean diet, suggests that subjects with a better olfactory sensitivity are also those that are best suited for a particular type of diet. The Mediterranean diet is generally recommended as the most suitable to improve the metabolic status [88,89] and reduce cardiovascular risk [90]. Indeed, MD is known to promote good health [91] due to several beneficial effects it provides against oxidative stress, low-grade inflammation, and gut microbiota which are strong contributors for the development of non-communicable diseases (NCDs) such as cardiovascular, metabolic, neurodegenerative diseases, and cancer [92]. The health benefits of MD are linked to its plant-based composition and the richness in fiber, vitamins, and minerals of the vegetable unprocessed foods and monounsaturated fats typical of this dietary model [62,93]. Nonetheless, despite the numerous and well-known healthy properties, plant foods, especially if consumed without seasoning, are commonly perceived as poorly palatable so that a low olfactory sensitivity could be a reason to limit or exclude them, preferring instead highly palatable foods rich in simple sugars or saturated fats [94], generally ultra-processed and hypercaloric, which also affect the reward system [95]. According to the energy balance model, a dietary pattern based on the overconsumption of high-density and poor nutrient foods, especially if associated with a sedentary lifestyle, is one of the main drivers of the obesity pandemic [96], and metabolic diseases [97]. Moreover, ultra-processed food, particularly added with sugar, plays an essential role in the development of NCDs [98]. Indeed, beyond the effect on weight loss, a healthy diet is a key for cardiovascular prevention [99,100,101], although a long-term slimming is not often achieved [102].

Considering that obesity is a complex disease and has multiple metabolic comorbidities such as hypertension, dysglycemia, dyslipidemia, and a resulting metabolic syndrome [99,100,101], the second objective was to evaluate the possible correlation between the olfactory scores obtained by patients and their values of systolic blood pressure, fasting plasma glucose, HDL and triglyceride levels. The results we obtained revealed an inverse correlation between TDI, T, D, and I olfactory scores on the one hand, and both systolic blood pressure, fasting plasma glucose, and triglyceride levels on the other, while no relationship was found with plasma HDL levels. These results confirm those of previous studies reporting that subjects with hypertension, diabetes/dysglycemia, and hypertriglyceridemia show a significantly higher prevalence of olfactory dysfunction [103,104].

Taken together, these results show the presence of correlations between smell, metabolic alterations associated with obesity, and eating habits. Therefore, the ultimate goal of our study was to try to understand how all these factors are connected to one another, what is the strength of connections, and whether there are any differences related to sex, through the Artificial Neural Network and the semantic connectivity map Auto-Contractive. By exploiting the functional distances within the entire spectrum of variables, this methodology has highlighted the underlying scheme of connections among variables. These analyses show a strong connection (0.98) between obesity and hyposmia, confirming and reinforcing previous findings on the inverse relationship between olfactory scores and BMI [41]. Furthermore, the semantic maps highlight a strong and direct connection between obesity and elevated systolic blood pressure, elevated blood glucose and triglyceride levels, and low plasma HDL levels. Given the direct connection between obesity and these metabolic alterations and between obesity and hyposmia, the obesity factor seems to act as a bridge between hypertension, dysglycemia and dyslipidemia, and olfactory dysfunction, in agreement with the inverse correlations we found. It has been suggested that in individuals with increased body weight, the circulating levels of orectic and anorectic peptides such as ghrelin, leptin, and insulin are modified, thus decreasing olfactory sensitivity [20,44,45,83]. An impairment of the olfactory function could modify the eating behavior and the choice of foods to compensate for the decreased gratification that occurs with eating due to sensory change [80,105]. Since people with hyposmia tend to prefer saltier and spicy foods, sweets and refined sugars, and a high-fat diet over fruits, vegetables, and sour and bitter foods [5,106,107,108], olfactory dysfunction can lead to overeating of high-calorie foods and weight gain [20,109,110], and a high-fat diet can worsen olfactory impairment due to the pro-inflammatory state that characterizes a high-fat diet [111]. A study on mice fed a fat diet showed that the number of olfactory neurons and their axonal projections were reduced; the Authors suggested that fat diet caused an increase in the degree of cell death, responsible for the reduced connections between epithelium and olfactory bulb, with consequent impairment of olfactory abilities such as discrimination and identification of odors [112].

Another controversial aspect linked to the olfactory function concerns the possible differences related to sex; some studies report a higher prevalence of olfactory dysfunction in males, while others do not observe any difference [26,27,113,114]. The semantic connection maps we obtained show a direct and strong connection between olfactory dysfunction and males, while females are directly connected with a normal olfactory function. These results, obtained with statistical methods of the latest generation, support those relating to the different distribution between males and females classified by their olfactory status. In fact, we found that the number of normosmic males for general olfactory function and for the identification of odors is significantly lower than that of females. In general, these findings support the idea that females perform better than males. The reasons can be multiple: endocrine factors (such as fluctuations associated with the menstrual cycle and circulating estrogen levels) [31,115,116], social factors (females show more attention and familiarity with odors) [28,29,117], and cognitive factors (sex-related differences were highlighted in episodic olfactory memory in favor of females) [24,29]. Furthermore, a recent study has highlighted the involvement of genetic factors in the sex-related differences in olfactory function (such as a polymorphism in the Kv1.3 gene) [32]. We assume that, in addition to the aforementioned reasons, an important role may be played by the higher adherence to the Mediterranean diet presented by females. In fact, not only did the Pearson test results show a positive correlation between the olfactory scores and the Mediterranean diet score, but the connectivity maps also showed a direct and very strong connection (0.97) between females and their adherence level to the Mediterranean diet. Instead, no correlation or connection was found in the case of males. This could be due to the fact that the male sample size is small compared to the female one, limiting the possibility of visualizing any connections, especially if these are weak, and this represents a limitation of the study. Further studies will therefore be needed to confirm these correlations and connections and, in particular, to try to bring out others that have not yet been found.

In conclusion, the mechanism that we propose could be the following: the better olfactory performance of females for the reasons previously reported can lead to a greater adherence to the Mediterranean diet, known to be the healthiest dietary model [118,119]; this seems to prevent the onset of dyslipidemia (as demonstrated by the direct and strong connection (0.98) between females and normal triglyceride levels). Consequently, the pro-inflammatory state that leads to the destruction of the olfactory neurons and their connections with the olfactory bulb cells would not occur and therefore a normal olfactory function would be maintained.

## Figures and Tables

**Table 1:** Anthropometric, clinical, nutritional, and olfactory characteristics of the whole sample and divided by sex.

Sex	F	M
N	51	17
Age (years)	57.69 ± 20.8	50.59 ± 3.73
Height (m)	1.56 ± 0.01	1.71 ± 0.02
Weight (kg)	83.19 ± 2.65	97.94 ± 4.55
BMI (kg/m^2^)	34.09 ± 0.95	33.59 ± 1.45
WC (cm)	108.21 ± 2.15	111.62 ± 2.45
S-BP (mmHg)	125.28 ± 2.19	129.53 ± 3.15
D-BP (mmHg)	78.82 ± 1.51	81 ± 2.20
FPG (mg/dl)	97.04 ± 2.64	103.47 ± 6.03
HDL-C (mg/dl)	59.77 ± 2.17	50.19 ± 3.44
TG (mg/dl)	108.22 ± 7.49	102.81 ± 13.43
MDS	30.22 ± 0.70	28.35 ± 1.50
TDI	26.96 ± 0.78	24.29 ± 1.40
T	5.86 ± 0.34	4.94 ± 0.57
D	10.24 ± 0.42	9.59 ± 0.73
I	10.86 ± 0.33	9.77 ± 0.43

WC: Waist Circumference; S-BP: Systolic Blood Pressure; D-BP: Diastolic Blood Pressure; FPG: Fasting Plasma Glucose; HDL-C: High-Density Lipoprotein-Cholesterol; TG: TriGlycerides; MDS: Mediterranean Diet Score; TDI: composite score as the sum of results for Threshold, Discrimination, and Identification Olfactory Test; T: odor Threshold score; D: odor Discrimination score; I: odor Identification score.

**Table 2:** Distribution of patients of the whole sample divided by sex (females n = 51, males n = 17) and classified as individuals with overweight or obesity based on their BMI (Kg/h^2^).

Variable	Sex	
BMI Status	F (%)	M (%)
Overweight	16 (31.37)	6 (35.29)
Obesity	35 (68.63)	11 (64.71)

**Table 3:** Distribution of patients of the whole sample, and divided by sex (female n = 51, male n = 17) showing hypertension, hyperglycemia, abnormal levels of high density lipoprotein (HDL) and triglycerides (TG), metabolic syndrome (MS), and lower adherence to the Mediterranean Diet (MDS) based on their clinical, metabolic and nutritional data.

	Sex	
Variable	F (%)	M (%)
Hypertension	29 (56.86)	12 (70.59)
Hyperglycemia	15 (29.41)	7 (41.18)
Low HDL	15 (29.41)	2 (11.75)
High TG	11 (21.57)	5 (29.41)
MS	20 (39.22)	7 (41.18)
MDS (<30)	21 (41.18)	9 (52.94)

**Table 4:** Distribution of patients classified as normosmic or hyposmic based on their overall TDI olfactory status and singly for their Threshold (T), Discrimination (D), and Identification (I) olfactory status.

Variable	Olfactory Status	n (%)
TDI	Normosmic	24 (35.29)
	Hyposmic	44 (64.71)
T	Normosmic	48 (70.59)
	Hyposmic	20 (29.41)
D	Normosmic	40 (58.82)
	Hyposmic	28 (41.18)
I	Normosmic	34 (50)
	Hyposmic	34 (50)

**Table 5:** Distribution of female and male patients classified as normosmic or hyposmic based on their overall TDI olfactory status and separately for their Threshold (T), Discrimination (D), and Identification (I) olfactory status.

	Group	F	M	*p*-Value
Variable	Olfactory Status	n (%)	n (%)	
TDI	Normosmic	23 (45.10)	1 (5.88)	0.003
	Hyposmic	28 (54.90)	16 (94.12)	
T	Normosmic	35 (68.63)	13 (76.47)	0.538
	Hyposmic	16 (31.37)	4 (23.53)	
D	Normosmic	29 (56.86)	11 (64.71)	0.569
	Hyposmic	22 (43.14)	6 (35.29)	
I	Normosmic	29 (56.86)	5 (29.41)	0.017
	Hyposmic	22 (43.14)	12 (70.59)	

*p*-Value derived from Fisher’s Exact Test. Females (n = 51), males (n = 17).

## Data Availability

The data presented in this study are available on request from the corresponding author. The data are not publicly available due to restrictions (e.g., privacy or ethical).
